# Men’s sheds as an alternative healthcare route? A qualitative study of the impact of Men’s sheds on user’s health improvement behaviours

**DOI:** 10.1186/s12889-021-10585-3

**Published:** 2021-03-20

**Authors:** Danielle Kelly, Artur Steiner, Helen Mason, Simon Teasdale

**Affiliations:** Yunus Centre for Social Business and Health, Glasgow Caledonian University, M201 George Moore Building, Cowcaddens Road, Glasgow, G4 0BA UK

**Keywords:** Men, Health engagement, Men’s sheds, Healthcare, Public health

## Abstract

**Background:**

Men’s health is a globally underrepresented area of research and policy. With men facing numerous healthcare barriers, there are calls for more ‘male friendly’ approaches to health improvement that take into consideration differing male behaviours and attitudes towards health. Men’s Sheds are community-based organisations delivering practical and social activities that encourage positive health behaviours. While Sheds have been recognised for their health and wellbeing benefits to men, research has yet to explore the impacts of Sheds on male health improvement and their potential role as a preventative gendered public health measure.

**Methods:**

The study used in-depth interviews with 62 Shed members from five Sheds to investigate the impacts of Shed activity on the health improvement behaviours and attitudes of Shed users. Findings from the qualitative study were used to propose a set of pathways in which Sheds activity led to positive health engagement.

**Results:**

The proposed pathways suggest that there are many different and interlinked ways in which Shed activities can impact on the health behaviours and attitudes of Shed users. Through participation in various practical and social activities in an inclusive environment, Shed users reported increases in their health seeking behaviours, improved perspectives on and management of their personal health, and an increased ability to overcome illness and recover.

**Conclusions:**

Where male friendly health provision has been lacking, this study suggests how Men’s Shed activities can provide positive male health outcomes, often in unexpected and non-obvious ways. In particular, the proposed visual pathways are important to inform policymakers and practitioners of the ways in that Sheds may contribute to engaging men in health improvement practices and increase their health knowledge. This study also provides a structure from which further studies can measure and evaluate Shed health impacts.

## Background

Men’s health outcomes are globally problematic and an underrepresented area of public health literature and policy [1, 2]. Studies have shown that men are more susceptible to illness and injury, as well as having a lower life expectancy than women [3, 4], with top causes of death in men found to be heart disease, cancers and suicide [5]. Men are more likely than women to engage in risk behaviours, such as excessive alcohol, smoking, and dangerous driving [6, 7], have a poorer diet [8], and be less conscious of risks associated with their weight [9]. Yet, men are also less likely to access formal healthcare or engage in health improvement behaviours [1, 10].

Challenges to male health improvement are inextricably linked to men’s behaviours and attitudes towards their health [7]. Commonly, health behaviours and attitudes are linked to gender norms where men are viewed as physically tough and resilient in the face of adversity, therefore taking care of their health or seeking support may be seen as a weakness or vulnerability [7, 11]. For instance, men have been found to be less inclined to seek help for mental health issues like depression, and are more prone to hiding their feelings from friends and family for fear of appearing fragile [12, 13]. It has also been observed that men, due to social constructs, tend to be less proactive in sourcing health information and advice than women, and less likely to recognise symptoms of potentially serious health problems [14, 15]. In many cases, men avoid dealing with practical barriers to accessing healthcare, such as GP opening hours or booking systems [1]. As a result, men can often be classed as a ‘hard to reach’ group for preventative healthcare [16].

With presented challenges in mind, there have been calls for more ‘male friendly’ approaches to health improvement; for example, the provision of healthcare in informal leisure venues attended by men, and the use of healthcare language suitable for male audiences [1, 17]. While studies have provided evidence of gender-based preventative measures for the health and wellbeing of women, very few studies describing male specific interventions exist [9, 18, 19]. Nonetheless, what has been highlighted is the value of targeting men in their communities, and allowing them to be involved in their development and operation from the outset at a grassroots community level [19]. Targeted ‘male friendly’ approaches allow interventions to be adaptive to the needs of men from varied ages, backgrounds and abilities, and health-related activities to be contextually appropriate [20]. As such, there is a need for policymakers and practitioners to recognise that strategies are required that consider differing behaviours and attitudes of men to improve their effectiveness and accessibility [2, 10].

The community-based model of Men’s Sheds has been highlighted as a potential space for men to engage in positive health behaviours in an informal and accessible environment [21, 22]. Men’s Sheds (referred to as Sheds hereafter) are organisations that originated in Australia in the 1990s in response to increasing concerns about men’s health [23]. However, they have seen growth over the past decade, predominantly in Western countries, such as the UK, Denmark and Canada. They provide opportunities for men to take part in meaningful practical and social activity, often in a workshop environment, that encourages social support and interaction with other men [24, 25]. Most notably, Sheds are spaces where activities are often tailored to the specific health and social needs of local men within community contexts [21]. Such spaces are viewed as important for men who might be reluctant to access formal public healthcare, including those marginalised through mental health issues and negative life experiences [26, 27]. They are also found to be particularly valuable to older men as a way of maintaining friendship networks, feelings of self-worth and a sense of belonging after retirement or during unemployment [22].

Studies of Sheds have outlined the impacts of Shed activities on aspects of user’s health and wellbeing, such as decreased levels of depression and increased confidence [28, 29]. However, in a recent review of Men’s Shed literature, Kelly et al. [30] identified that evidence is lacking of the pathways to which Sheds can impact on men’s attitudes and behaviours towards health improvement. Such studies are required to enable policymakers and health practitioners to understand the potential role of Men’s Sheds as a preventative gendered health measure.

Considering existing knowledge gaps, and drawing on primary data from five Sheds in Scotland, this paper aims to provide a contribution to evidence of the ways in which Sheds impact on the health behaviours and attitudes of their users. To address this aim, we build on the work of Kelly et al. [30] and use primary qualitative data to propose a set of pathways by which Sheds can provide an alternative way of engaging men with health improvement activities. Presented findings generate new knowledge in an under-researched, yet important, area of public health literature and policy. Our discussion provides implications for wider health policy and practitioners, and suggest how the Shed intervention may fit into current health and social care practice to tackle challenges of ‘hard to reach’ men and reduce some of the described inequalities.

## Methods

To identify the ways in which Sheds can impact on the health behaviours and attitudes of their users, an in-depth qualitative study was undertaken with Sheds users from across Scotland.

### Study context

Sheds in Scotland have developed quickly, with recent figures showing their numbers have grown with the first Shed opening in 2013, to approximately 190 registered with the Scottish Men’s Shed Association in 2020. Sheds are operating across varied contexts, both geographically and demographically, and with the existence of Sheds that work both independently of and in partnership with the state. Although Scottish policy documents recognise Sheds’ potential to tackle social isolation and loneliness [31], research on the wider health benefits of Sheds in this context is lacking with only a handful of studies in existence in the UK [32, 33].

### Recruitment and sample

A list of 98 registered Sheds was provided by the Scottish Men’s Shed Association (SMSA), a national support agency for Sheds. From this list, 15 Sheds were shortlisted based on the inclusion criteria of (i) having at least 20 members, (ii) having a fixed space where members meet and to provide a location for data collection to take place (iii) and, having no less than fortnightly activity to ensure that members were in a position to talk about the health and wellbeing impacts of their Sheds. From the 15 Sheds, five were selected based on their demographic and geographic characteristics (Table 1) to ensure a variation of Sheds from both rural and urban areas, as defined by the Scottish Government urban/rural classification [34], and deprived and affluent areas, as defined by the Scottish Index of Multiple Deprivation [35]. The five Sheds were also selected based on their availability and willingness to take part in the study.

**Table 1:** The characteristics of Sheds included in the study

	Shed Characteristics
**Shed 1**	100+ members Urban location Mid-level deprivation
**Shed 2**	30+ members Remote/ Rural location Affluent area
**Shed 3**	80+ members Urban location Deprived area
**Shed 4**	20+ members Urban location Deprived area
**Shed 5**	20+ members Urban location Affluent area

Initial Shed member contacts were sought to facilitate access to each Shed and an introduction to all Shed members. From this, snowball and convenience sampling techniques were adopted to recruit members for interview [36]. The demographics of participants included in the study are shown in Table 2.

**Table 2:** Participant demographics

**Shed 1**	**Age Range**	**No. of Shed members**	**Employment Status**	**No. of Shed members**
	50–60	1	Retired	11
	60–70	5	Working	0
	70+	6	Sick/ disabled	1
**Shed 2**	**Age Range**		**Status**	
	50–60	1	Retired	12
	60–70	7	Working	0
	70+	6	Sick/ disabled	2
**Shed 3**	**Age Range**		**Status**	
	40–50	1	Retired	14
	60–70	4	Working	0
	70+	10	Sick/ disabled	1
**Shed 4**	**Age Range**		**Status**	
	20–30	2	Retired	7
	40–50	2	Working	1
	50–60	1	Sick/ disabled	4
	60–70	5		
	70+	2		
**Shed 5**	**Age Range**		**Status**	
	60–70	5	Retired	9
	70+	4	Working	0
			Sick/ disabled	0
**Total**	**Age Range**		**Status**	
	20–30	2	Retired	53
	40–50	3	Working	1
	50–60	3	Sick/ disabled	8
	60–70	26		
	70+	28		

As shown, the majority of study participants were over the age of 60 years and retired. The exception was Shed 4 which had younger members who were unable to work due to sickness or disability.

### Data collection and analysis

Over the course of our study, 62 in-depth interviews were conducted with Shed members. The interviews allowed the researcher to probe areas of health and wellbeing in a flexible manner, and to employ a conversational style of interviewing that was best suited to informal Shed environments [37]. The topic guide was informed by the literature in the field and focused on the health and wellbeing impacts of Sheds on their users, but allowed for the exploration of other related themes. This included questions on the impacts of specific Shed activity on the health and wellbeing of users; and changes in participant’s behaviours or attitudes towards their health and wellbeing as a result of attending a Shed. The study adopted the World Health Organisation definition of health as *‘a state of complete physical health, mental health and social wellbeing, and not merely the absence of disease or infirmity’* ([38] :1). Physical and mental health were described to participants as relating to the status of the individual participant, whereas social wellbeing was described in terms of the individual’s relationship with others, including those within their Shed group, their community and wider society [39]. This provided clarity for participants on the meaning of health and wellbeing, and allowed for a deeper exploration of each aspect of men’s health. Ethical approval for this study was granted by Glasgow Caledonian University ethics committee and all methods were carried out in accordance with relevant guidelines and regulations.

Before commencing each interview, participants were given time to read the participant information sheet that outlined the nature of the study, and informed consent was received. Interviews took place at the Sheds premises in a private area away from other Sheds activities to ensure that participants felt comfortable to discuss topics related to their health and wellbeing. Interviews lasted 30–60 min and were audio-recorded and stored on a password encrypted folder and laptop. Interviews were transcribed and analysed using the qualitative data analysis software NVivo. Data was first organised using descriptive coding techniques to identify topics related to the aims of the study, such as physical health, mental health and social wellbeing. Secondly, each topic was explored in further detail and broken down into headings and sub-headings, and duplicate codes were merged [40].

### Development of logic model

The qualitative data underpinning the coding framework provided evidence on the ways in which attending a Shed could impact on the physical and mental health and social wellbeing of Shed users. The next stage of the analysis was to identify *how* and *why* this was achieved. Such information is useful to inform policymakers of the various routes to which Sheds inputs can lead to improved health, in order to gain a better understanding of where support may be required. Further, as stated by Kelly et al. [30], it can provide a structure for future measurement and evaluation of Shed impacts, and to inform the development and delivery of community-based health activity.

**Fig. 1:**
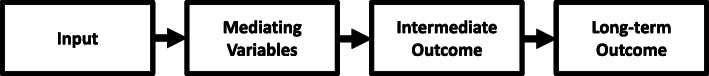
Mapping of data from inputs to outcomes

Accordingly, analysis focused on identifying data that described links between Shed activities and the processes that led to impacts on health behaviours and attitudes. Data was then mapped into pathways using the structure presented in Fig. 1.

**Fig. 2:**
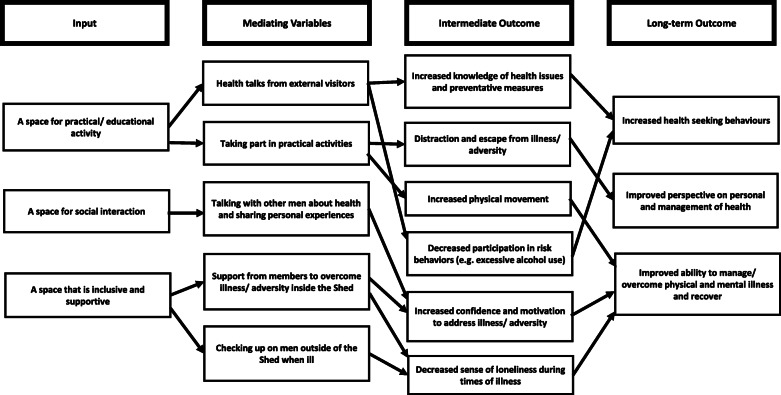
A logic model of proposed health improvement outcomes from Shed activities

The input was defined as the activity or resource that was provided by the Shed (e.g. social activities). Mediating variables were defined as the relationship between the input and the outcomes; for example, the input of social activities led to increased social interaction between Shed members, which then led to positive health outcomes. Intermediate and long-term outcomes were defined as the short and long-term effects of the Shed activity on Shed member’s health behaviours and attitudes. Building on the conceptual framework presented by Kelly et al. [30], the analysed data was then developed into a logic model (Fig. 2), providing a visual representation of proposed pathways to the health improvement of Shed users (Fig. 2).

## Results

### Key findings

Of the 62 Shed members interviewed, 40 reported having an existing illness or injury^1^ diagnosed by a health professional, whilst the remaining 22 members without diagnosed health issues commented on the ways in which the Shed might help to sustain their future health and wellbeing.

As previously shown in the work of Kelly et al. [30], the qualitative data from this study also indicated that the Sheds provided three specific inputs that directly impacted on the ability of members to improve their health. These were (1) ***Practical/ educational***: space to take part in practical activities, where skills are learned and shared; (2) ***Social/ interactive***: space to socialise and interact with others and form social relationships and networks; (3) ***Inclusive/ supportive***: an informal and flexible space where men of all backgrounds are included and can gain social support and share experiences in a safe ‘male-friendly’ environment. Findings will now focus on each of these inputs, and describe the pathways that led to improved health behaviours and attitudes in Shed users.

#### Practical/ educational aspects

Members with reported diagnosed health issues found that taking part in practical activities, like woodwork, provided a distraction and escape from illness or adversity in their lives, helping them to relax and *‘switch off’* (Shed 1 Member).

*‘I’ve got IBS and diverticulitis and stress is a big part of keeping that under control … the Shed helps me with that, because these are all things that go when you’re concentrating on something else … It’s a big distraction, because I do worry about my health.’ (Shed 2 member).*

*‘I’ve got Alzheimer’s, so being here helps me on that, because it gives me something to put attention on and it just seems to help my mind. My memory, I can remember things from here … and can get a bit of reality.’ (Shed 5 member).*

Having a distraction and sense of escape provided them with increased feelings of strength to manage or cope with symptoms of illness, such as pain or memory loss. Across members with and without reported health issues, taking part in practical workshop activities was also found to increase member’s physical movement.

*‘I was limping so badly … the pain in my feet and mobility was so restricted … So I’ve found my strength is gradually building up and the stamina is building up too … because I’m spending more time on my feet and doing things so your keeping occupied and not necessarily standing still in one spot.’ (Shed 5 member).*

Taking part in physical activity was particularly important for those with physical health issues, such as arthritis or trauma injuries, to help them manage symptoms and aid recovery.

Three of the five Sheds invited educational speakers, such as health workers and nurses, to talk to the men about health issues such as strokes and bowel cancer. This included talks on specific male health problems, such as prostate and testicular cancer.

*‘Two doctors came down and they spoke about strokes and how to avoid them, and people who normally don’t speak were asking questions because they don’t want a stroke … So now they know a lot.’ (Shed 1 member).*

As a result of receiving these educational health talks, a number of Shed members reported increased willingness to improve their health-seeking behaviours. This was particularly relevant to members without any reported diagnosed health issues as a way to educate themselves on preventative lifestyle measures.

*‘Because we’ve had people in talking about health, it sort of triggers you to sort of think well I need to start looking after myself, and I have done that … I’ve cut out lots of sugar, I don’t drink anymore, things like that.’ (Shed 1 member).*

*‘We had the doctors in the other day there … so I actually did a lot more research into that on the internet … it’s actually made me think about my health more … I have lost a stone and a half in the past couple of months.’ (Shed 2 member).*

Most notably, talking to health professionals, as well as other Shed members about sensitive and personal issues in a supportive environment led to decreases in excessive alcohol use of members that had addiction issues.

*‘I’m not even drinking a quarter of what I used to … during the day I don’t touch it, but before actually I was going through a bottle and a half in a week before … So, if the Men’s Shed wasn’t here, I wouldn’t be here to tell the story … I’d have drunk myself to death.’ (Shed 2 member).*

*‘There are a few guys in there already have similar problems (with alcohol) to me and I can see the benefit in them, they can see the benefit in me. So, we work with each other and talk to each other, so everybody benefits.’ (Shed 4 member).*

Further, as a result of educational health talks some members reported decreasing their levels of tobacco intake as a way to preserve their ability to attend the Shed in the future, and to enable them to take part in Shed activities that required physical exertion. Resultantly, members felt that opportunities to talk to health professionals in their own comfortable Shed environment, rather than attending a formal health care service, were a vital form of harm prevention.

#### Social/ interactive aspects

In providing space for socialising and interacting, the Sheds fostered an environment where men could talk to each other about their life experiences, including personal issues and health concerns. Shed members reported that as well as interacting in designated social areas in the Sheds, working *‘shoulder to shoulder’* (Shed 1 member) with other men in a workshop environment provided opportunities to converse. This was often a more comfortable option for those who lacked confidence or ability to sit down and talk face to face with other men.

For many, the social aspects of the Sheds were more important than the practical, particularly for those not interested in workshop activities.

*‘Like they say, a problem shared is a problem halved because I’ve met a few guys who’ve gone through much more strokes than I have and they’ll say, “Don’t worry”, and I’ll toss them a wee bit of the benefit of my advice to them of my personal circumstances.’ (Shed 2 member).*

Sheds were found to offer a socially acceptable ‘*safe space’* (Shed 3 member) for men, to talk openly about their health problems, in particular those with mental health concerns, where opportunities may have not have previously existed in typical male environments, such as bars and sports clubs.

*‘In a drinking club, it’s not discussed. At a dart’s club, things are not discussed. Here, you could if you wanted to, open up and you’d grab somebody and tell them what struggles you have and I think everybody here would give you a bit of help, guidance or support.’ (Shed 4 member).*

Further, for those with reported diagnosed health issues, having opportunities to talk to other members about their concerns was found to improve their perspective on their own health.

*‘I come in here and I talk to some of the other guys in here, and see they’re still going with things that are 10 times worse than mine, and it kind of changes your viewpoint.’ (Shed 1 member).*

Sharing experiences of illness and adversity with other Shed members gave individuals an increased confidence and motivation to not let their illness dictate their lives and to aid their recovery.

#### Inclusive/ supportive aspects

All of the Sheds aimed to provide a space that was inclusive and supportive, and where men of all backgrounds are welcomed. For example, two of the Sheds introduced an induction system whereby new members were partnered with an existing Shed member to help them to integrate. In creating an inclusive space, members with reported health issues found that they had gained a great deal of social support from other members to help them to overcome illness or adversity. Support also extended outside of Shed hours with members providing regular phone call or visits to check up on those at home or in hospital.

*‘When I was off and was sitting in the house I used to get a phone call every day saying ‘are you wanting to see anybody?’ So then someone from the Shed would come up and it was great because you think ‘they’re thinking about me’. So that builds your confidence up, so the quicker I get better the quicker I can get in among it again.’ (Shed 3 member).*

Gaining such support from members was found to increase men’s confidence and motivation to address health issues that had caused physical or mental setbacks.

*‘I couldn’t speak to anybody when I came out the coma (after brain injury) and basically I didn’t know who my wife was, didn’t know who my children were, I was harming myself … but I got the help that I needed from this Shed. They were calming me down, putting me into meetings and saying, “If you don’t know, just ask”, and I did.’ (Shed 4 member).*

In particular, men reported that in sharing experiences and gaining support and guidance from other men who had been through similar adversity they felt less alone with their illness, which aided their ability to recover.

*‘There’s other folk here who’ve been through the exact same things as me and they can tell you the exact way to get through it, you’re not just dealing with it yourself.’ (Shed 2 member).*

*‘I’ve been diagnosed with this non-benign tremor. So, having a chat with the other boys, they’ve got something similar, so you’re not on your own so you just get on with it. Toughen up sort of idea.’ (Shed 2 member).*

Gaining social support from other men was found to be particularly important for men with reported diagnosed mental health issues such as depression and anxiety, as a way to open up in a safe and secure male environment free of stigma.

*‘In the British culture men don’t talk much about mental health, they don’t want to show their weakness … I’ve started talking to people who’ve been through depression or know where I’m coming from … .so I find that its actually helpful for me, but I’m sure others also benefited as well from talking to me.’ (Shed 5 member).*

*‘Men need somewhere to discu****s****s things freely … Men don’t tend to do that, they bottle it up, they don’t want to ask you about this, they don’t want to ask you about that, whereas here over a cup of tea they’ll open up’ (Shed 5 member).*

Shed members without professionally diagnosed mental health issues also felt that they were able to overcome symptoms related to anxiety and depression simply through gaining support from other Shed members, rather than relying on professional health services.

## Discussion and conclusion

Drawing on the data described, a logic model was developed to provide a visual representation of proposed pathways from the input of Shed activities to the health improvement of Shed users (Fig. 2).

What this models shows is a complex system of pathways whereby there are many different and interlinked ways in which Shed activities can impact on the health behaviours and attitudes of Shed users. In particular, it demonstrates some of the unexpected and non-obvious ways in which activities can have positive health outcomes. For example, men may attend a Shed simply to partake in practical activities, however, this may also lead to unexpected improvements in their ability to cope with adversity. Such visualisations are important to inform policymakers and practitioners of the ways in which initiatives like Sheds, that are not directly delivering a health service, can contribute to engaging men in health improvement practices and increase their health knowledge. As causal links can only be proposed at this stage, this logic model also provides a structure from which further studies can measure and evaluate Shed health impacts. With this in mind, research would also benefit from further quantitative investigation, for example, the use of validated health tools to measure changes in the health of Shed users after continued involvement in activities, especially in those with diagnosed health conditions.

As highlighted by Kelly et al. [30] previous studies of this kind have been missing, therefore, this study is the first of its kind to use primary empirical data to propose pathways from Shed inputs to health outcomes resulting from changes in health attitudes and behaviours. While presented findings from one qualitative study are limited in their generalisability, they provide a valid contribution to the wider growing body of knowledge on the health impacts of Sheds, and the role and effectiveness of gender-specific health initiatives.

As shown in the literature, ‘male friendly’ approaches to health improvement are lacking, and interventions are required that consider differing male behaviours and attitudes [1]. This empirical study has shown that Shed activities can help members with reported diagnosed health conditions to feel as though they can overcome illness and adversity, and promote those without existing health issues to engage in preventative health improvement measures. The creation of an inclusive and supportive ‘safe space’ where men feel relaxed and willing to discuss health problems is key to this process. The ‘Shed model’ is found to cater particularly well for those reluctant to engage with more formal public healthcare services, especially for mental health concerns [12, 13]. The informal and flexible nature of Sheds means that their activities can be tailored to the specific needs of these individuals, unlike more structured formal state alternatives. What this study also shows is that men can effectively access professional health advice through educational talks from health visitors, without having to leave their ‘safe’ Shed environment.

While recognising the health benefits that Sheds deliver, policymakers and health and social care practitioners must consider that Sheds are not formal service providers, nor do they house individuals with professional experience of dealing with physical and mental health issues. Sheds are volunteer-led organisations with predominantly older and retired members, set up to provide leisure activities on an ad hoc basis. For these reasons, it is likely that Sheds could only provide a complementary, rather than an alternative, route for male health improvement that exists alongside formal public healthcare services. Therefore, policymakers and practitioners must find novel ways to co-exist and work in partnership with such organisations to ensure a wide reach when planning male health interventions for those with and without existing health conditions. Examples of such partnerships may include sign-posting or social prescribing of men [41] to Sheds by health professionals, and continued support for regular access to preventative health education from professionals (i.e. health talks). However, as Sheds are volunteer organisations, this would require an appropriate level of directed support to enable the continued delivery of regular activities to meet the specific health needs of men within communities.

## Data Availability

The datasets used and/or analysed during the current study available from the corresponding author on reasonable request.

## Footnotes

1 Reported diagnosed health issues across all Shed included physical illness/injury; cancer, strokes, heart conditions, brain injuries, kidney failure, skin conditions, age related mobility issues (arthritis/ osteoporosis), and pain from accident/trauma. Also illness related to mental health and mental ability; mental breakdowns (psychosis, PTSD), alcohol addiction, Alzheimer’s/ Dementia, depression and anxiety.
